# *Innovation* focus in 2025

**DOI:** 10.1016/j.xinn.2025.101223

**Published:** 2025-12-10

**Authors:** The Innovation Editorial Team, China

## Main text

**Figure 1:**
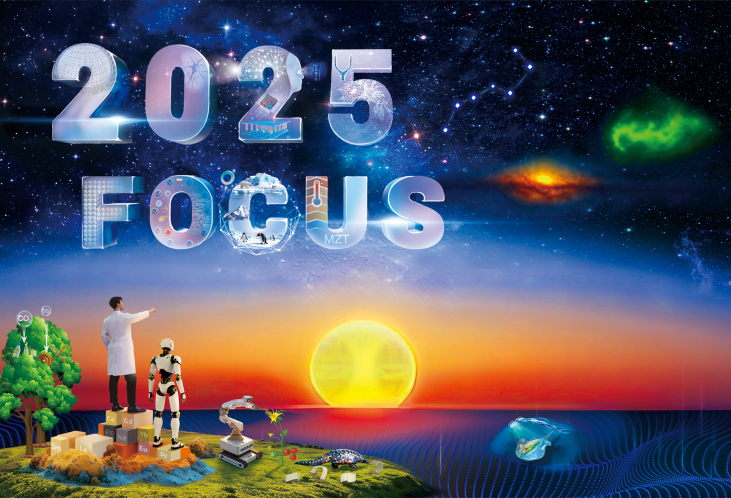
*Innovation* focus 2025

Review of scientific breakthroughs in 2025, from large scientific facility innovations to united climate threshold warnings, from biomass carbon sink solutions to functional materials and rare earth advances, from integrated circuits enabling hardware to AI-bio-health-robotics integration, and onward to personalized medicine and smart agriculture applications, …, by *The Innovation*’s editorial team (Figure 1).

## Smart agriculture: Shaping a new paradigm for agricultural research

Agricultural research is undergoing a profound paradigm shift, driven by smart agriculture. The development of an agricultural “digital brain” enables precise, full-chain monitoring, and intelligent decision-making, laying a resilient foundation for future food systems. As a core engine of smart agriculture, intelligent breeding is propelling the agricultural “chip”—seeds”—into a new era. Breakthroughs and refinements in synthetic apomixis allow for the permanent fixation of superior hybrid vigor, fundamentally transforming seed production models. Technologies such as polyploid breeding unlock new frontiers in crop genome design and *de novo* domestication, significantly accelerating precision breeding processes. Scientists pioneered the “crop-robot co-design” strategy, which deeply integrates biotechnology with information technology, giving rise to “robot-friendly” male-sterile exserted-stigma lines. The creation of the first intelligent breeding robot “GEAIR” (genome editing with AI-based robots), overcomes the efficiency and accuracy limitations of traditional manual pollination, leading the global breeding industry into an intelligent acceleration era. Looking ahead, smart agriculture will continue to reshape the mindset and methodological frameworks of agricultural research, steering the sector toward greater intelligence, sustainability, and eco-friendliness.

## Warning of temperature threshold breach: United in science

The 1.5°C warming target of the Paris Agreement is facing severe challenges, with 2024 marking the first year global average temperature rise exceeded 1.5°C relative to pre-industrial levels. Although global warming eased to 1.42°C ± 0.12°C in January–August 2025, the single-year breach has sent a strong signal, highlighting the urgency of globally coordinated climate action. Regrettably, the United States withdrew from the Paris Agreement once again in 2025, adding uncertainty to the multilateral climate governance system and complicating implementation of third round Nationally Determined Contributions. As the “outposts” of climate change, the “Three Poles of the Earth”—the Arctic, Antarctic, and Qinghai-Tibet Plateau—profoundly influence the global climate through atmospheric and oceanic connections. Meanwhile, technological means such as precise prediction based on complex climate networks are becoming crucial for addressing extreme weather. The World Meteorological Organization's “United in Science” slogan underscores technology's essential role in empowering low-carbon transitions and ecological protection for global climate adaptation and mitigation.

## Turning waste into carbon sinks: Lignocellulosic biomass-based materials driving the circular economy

Driven by global commitments to “carbon peaking and carbon neutrality” and “plastic restriction” targets, the development of lignocellulosic biomass-based carbon-sink materials has become a key direction for “carbon mitigation-pollution reduction innovation.” Constructing non-grain biomass-derived low-carbon or carbon-negative materials can reduce fossil resources dependence, alleviate plastic pollution, and convert agricultural and forestry residues into biodegradable bioproducts, achieving long-term biogenic carbon sequestration and value-added utilization of biomass. Recent advances highlight the potential of such materials. Microwave-assisted rapid hybridizing, for example, can rapidly turn waste paper and CO_2_ into high-performance paper plastics, offering a practical strategy for “paper substitution for plastics.” Another example is the direct conversion of woody biomass into a carbon-negative structural material—bio-strong-wood, through a green and efficient bio-assisted engineering strategy realizing zero waste discharge processing of woody biomass and net-negative carbon emissions across the entire life cycle. These biomass-based carbon-sink materials mark shift the materials field from traditional carbon-emission-reduction approaches toward genuinely negative carbon solutions. Their continued development will play a pivotal role in advancing the circular economy and supporting global sustainability goals.

## Biological health-emergence of new technologies and integration with AI

In 2025, innovative technologies are propelling biological research toward practical health applications. Antimicrobial resistance remains a major global threat. Researchers have pioneered a paradigm linking geometry, mechanics, and infection, providing novel insights into targeting physical microenvironment for anti-infective therapies. Organoid technology offers highly biomimetic, human-relevant models. A breakthrough single-chain-activating antibody resolves key bottleneck in organoid culture, enabling standardized production and clinical-grade uses. AI is transforming life sciences: OvaRePred, the world’s first personalized model for predicting ovarian function decline, exemplifies this shift. Virtual cells, constructed from vast single-cell omics data and large AI models, form digital twin systems that can accurately simulate real cellular dynamics. Looking ahead, integrated combining advanced molecular interventions, AI foundation models, biomedical imaging, and and multi-omics data will unravel disease mechanisms. This will usher biology into an era that is predictable, simulable, and engineerable.

## Integrated circuits: MZT new devices enabling the enhanced footprint and function integration

As Moore’s Law stalls, integrated circuit are shifting toward functional integration. transformative device, multifunctional multi-terminal zero-additional-resistor-process one-transistor with the novel channel electrode design architecture (MZT), integrates sensing, memory, and logic functions into a single silicon-compatible unit, laying a foundation for industrialization for in-memory computing (IMC) chips. In addition to traditional computing-in-memory (CIM) devices limited to analog current outputs for storage, MZT also enables digital voltage outputs for digital computation, realizing brain-like “perception-storage-computation”. In power-sensitive scenarios, MZT-based IMC chips and CIM chips demonstrate significant advantages. The brain-inspired chip Speck™, built on conventional CIM devices, set a commercial benchmark. Utilizing a spiking convolutional neural network architecture, this chip achieves milliwatt-level low-power operation. Furthermore, the thin-film transistor (TFT) technology is compatible with MZT. DNA microarray synthesizers based on the mature TFT technology have drastically reduced the cost of DNA synthesis, revolutionizing life sciences. Breakthroughs in functionality and area integration enabled by MZT are spearheading a semiconductor revolution across diverse disciplines.

## Robotics: The co-evolution of physical intelligence and embodied cognition

Robotics is embracing the transformative shift toward “embodied intelligence,” evolving beyond “pre-programmed automation” to a synergistic evolution of physical and cognitive intelligence. At the physical level, environmental coexistence biomimetic millirobots establish a new paradigm of “endogenous physical intelligence.” By leveraging smart materials they acquire an innate “sensing-deformation-adaptation” capability, transitioning from passive intervention to active adaptation in complex environments. At the cognitive level, Embodied Foundation Models adopt a scalable, data-driven engineering paradigm. Utilizing large-scale real-world data , they construct universal robotic learning platforms employing a “latent action space” interface. This bridges multimodal inputs with action outputs, facilitating generalization rooted in “learning by analogy” and reducing development thresholds. Ultimately, from the awakening of “physical instinct” to reconfiguration of “cognitive generalization,” robotics is accelerating its integration into society through the deep coupling of physical entities and digital intelligence.

## Novel functional metal-organic framework materials

Molecular structure is the key to new functional materials. In 2025, Susumu Kitagawa, Richard Robson, and Omar M. Yaghi won the Nobel Prize in Chemistry for pioneering metal-organic framework (MOF) synthesis and establishing a new precise MOF construction of. This year, MOF design and regulation ability has reached new heights. Researchers have proposed a “supramolecular docking” strategy to solve alkyl chain-containing molecules structure determination. The “energy carrier synergistic transport” concept aids MOF porous and functionalities design, while a “multi-scale structural disorder” strategy first endows MOFs with room temperature weak ferromagnetism. These strategies drive MOFs progress, bringing revolutionary advances to applications in emerging fields like energy storage and sensing.

## Precise regulation: Life science enters a new era of personalized medicine

Life science is undergoing a paradigm shift from observation and description to precise design and intervention: the DNA-writing-based programmable artificial transcription factor library (DIAL technology) achieves fine-tuned gene expression by programming the spatial structure of promoters; targeting “migrasomes” has successfully delayed brain aging; the “thromboinflammation-on-a-chip” has enabled the observation of the entire process of microvascular thrombus formation and dissolution, revealing the dual role of neutrophil elastase and validating the therapeutic window for thrombolytic drugs … These advances establish a comprehensive novel capability for precise regulation, from genes to intercellular communication to simulating pathologies. This represents more than a technical upgrade; it heralds a fundamental shift in medical paradigm, from generalized treatment to truly personalized therapy As our ability to mastery of molecular, cellular, and systemic biological processes deepens, personalized medicine is rapidly transitioning from a scientific vision into clinical reality.

## Large scientific facilities: The cornerstone and engine of basic research innovation

Several Large Scientific Facilities have been completed and are operating efficiently as scheduled. Experimental Advanced Superconducting Tokamak achieved a steady-state high-confinement plasma for a remarkable 1,066 s at 100 million°C, marking a new stage for China’s research on controlled nuclear fusion. The Five-hundred-meter Aperture Spherical Radio Telescope systematically explored the physical nature of black holes and observed a complex filamentary structure network dominated by supersonic turbulence within an interstellar gas cloud moving at ultrahigh speed in the Milky Way. The High Energy Photon Source project passed process acceptance, while the High Intensity Heavy-ion Accelerator Facility successfully completed beam commissioning. Besides, the Large High Altitude Air Shower Observatory uncovered mystery of cosmic ray energy spectrum, and the Jiangmen Underground Neutrino Experiment precisely measured the solar neutrino oscillation parameters. Furthermore, Chinese Academy of Sciences launched its international program for “Burning Plasma” and released the research plan for the Burning Plasma Experimental Superconducting Tokamak. Large scientific facilities have become the cornerstone and engine of basic research innovation, enabling humanity to deepen its understanding of the origins of universe, the composition of matter, and explore new pathways for sustainable energy development.

## Rare earths for a high-performance future

Rare earth elements are often called the “vitamins” of modern industry and are essential for carbon neutrality and advanced manufacturing. Today, rare earth research is shifting from empirical trial-and-error toward rational design enabled by precise atomic-scale control. In the new energy sector, permanent magnets—enhanced by grain boundary diffusion and microstructure optimization—are forging stronger “hearts” for wind turbines and electric vehicles. Similarly, catalyst based on “electronic-structural-dynamic” mechanisms have broken the activity single-atom activity limits, serving as “green engines” for carbon neutrality and hydrogen energy strategies. Crucially, rare earth materials are overcoming performance bottlenecks in critical infrastructure. Advances in nanoscale polishing slurries, ultra-fast scintillator, and heat-resistant alloys have ensured that integrated circuits, medical imaging devices, and aerospace components perform reliably even under extreme conditions. This progress marks a complete chain from fundamental research to practical application. Looking ahead, the integration of AI-driven research and green extraction will further support a modern, sustainable industrial system.

## Declaration of interests

The author declares no competing interests.

